# Pathologic Complete Response Achieved in Early-Stage HER2-Positive Breast Cancer After Neoadjuvant Therapy With Trastuzumab and Chemotherapy vs. Trastuzumab, Chemotherapy, and Pertuzumab: A Systematic Review and Meta-Analysis of Clinical Trials

**DOI:** 10.7759/cureus.39780

**Published:** 2023-05-31

**Authors:** Faizan Fazal, Muhammad Nauman Bashir, Maham Leeza Adil, Usama Tanveer, Mansoor Ahmed, Taha Zahid Chaudhry, Ali Ahmad Ijaz, Muhammad Haider

**Affiliations:** 1 Department of Medicine, Rawalpindi Medical University, Rawalpindi, PAK; 2 Department of Medicine, Holy Family Hospital, Rawalpindi, PAK; 3 Department of Surgery, Holy Family Hospital, Rawalpindi, PAK; 4 Department of Orthopedics, Holy Family Hospital, Rawalpindi, PAK

**Keywords:** docetaxel-trastuzumab-pertuzumab therapy, her2, her2+ breast cancer, neoadjuvant systemic therapy, pathologic complete response (pcr), randomized clinical trial, systemic chemotherapy

## Abstract

Patients diagnosed with human epidermal growth factor receptor 2 (HER2)-positive breast cancer require treatment upfront because of the aggressive nature of this type of cancer. Patients with early-stage HER2-positive breast cancer are usually treated with neoadjuvant therapy. This neoadjuvant therapy comprises targeted therapy and chemotherapy. Targeted therapy is given with trastuzumab. Pertuzumab is either administered or not with trastuzumab as a targeted therapy. This systematic review and meta-analysis aim to find out and compare the benefit achieved in terms of pathologic complete response (pCR) by adding pertuzumab to the neoadjuvant treatment regimen for early-stage HER2-positive breast cancer patients. Various databases were searched to find out relevant clinical trials. After going through PubMed, Embase, and Cochrane, three clinical trials were shortlisted for this systematic review and meta-analysis. These three clinical trials were double-armed. Pertuzumab was present in one arm while being absent in one arm to assess the benefit of adding pertuzumab in terms of pCR achieved. Data were analyzed using RevMan Web (Cochrane, London, UK). The odds ratio and 95% confidence interval were calculated for the outcome. The Mantel-Haenszel method and random effect model were used for analysis. The risk of bias in studies was evaluated using the Cochrane risk of bias tool for randomized controlled trials (ROB2). The summary statistics showed that the incidence of pCR was more in the experimental group (having pertuzumab) as compared to the control group (without pertuzumab) with an odds ratio of 2.10 (95% CI: 1.56-2.83) with I2 = 0%. In three double-arm trials, there were 840 participants, 445 in the experimental group and 395 in the control group. A total of 203 (45%) patients out of 445 in the experimental group achieved pCR, whereas 127 (32%) patients out of 395 in the control group achieved pCR. Through the results of this study, it can be concluded that the rate of pCR achieved was higher in that arm in which pertuzumab was present compared to the study arm in which only trastuzumab was given as targeted therapy. Thus, it can be suggested that pertuzumab be added to the neoadjuvant regimen for early-stage HER2-positive breast cancer patients. This would result in achieving a better pCR. And by improving pCR rates, the survival outcomes of patients can be significantly improved.

## Introduction and background

Breast cancer demands immense attention from both patients and care providers as it is the most common cancer occurring globally in human beings [1]. Due to improved screening techniques and infrastructure, the incidence of breast cancer is relatively more in developed countries [2]. On the other hand, the mortality due to breast cancer is more in the developing world, probably because of the diagnosis of cancer at an advanced stage and the unaffordability of treatment [3]. This is why around 60% of the total deaths due to breast cancer occur in the developing world [4]. Breast cancer is a heterogeneous disease with different clinical findings, treatment responses, and histologic types [5]. Receptors found in breast cancer cells are of particular importance. Estrogen receptors (ER) and progesterone receptors (PR) are hormone receptors in breast cancer cells. The relative presence or absence of these receptors in tumor cells defines the hormonal status of the tumor. The hormone receptor status of tumor cells determines the endocrinologic treatment strategy. In the majority of breast cancer patients, the ER and PR are overexpressed [6,7]. Endocrine therapy is given to breast cancer patients with ER/PR positivity. This therapy broadly includes ER modulators (e.g., tamoxifen), aromatase inhibitors (e.g., anastrozole and letrozole), and selective ER degraders (e.g., fulvestrant) [8]. Another receptor type found in breast cancer cells demands huge attention while planning treatment and deciding the prognosis of the disease; this is the human epidermal growth factor receptor 2 (HER2). HER2 is found to be overexpressed in 20% to 25% of breast cancers. HER2 is a proliferative signal and plays a crucial role in the uncontrolled division and proliferation of breast tumor cells [9]. The HER2 oncogenes (HER2, HER2/neu, and c-erbB-2) are found to be located on chromosome number 17 [10]. HER2-positive breast cancers are less responsive to chemotherapy and have proven to be more aggressive as compared to HER2-negative tumors [11]. The early-stage HER2-positive breast cancer is treated with targeted therapy and neoadjuvant chemotherapy. Trastuzumab is an anti-HER2 antibody. It is a form of targeted therapy. Research has shown that adding trastuzumab to the chemotherapy regimen results in a reduction of cancer recurrence rates and breast cancer-related mortality by a third [12]. Most women with HER2-positive breast cancer will receive one or more chemotherapy drugs plus trastuzumab, the anti-HER2 antibody. Many studies have shown that these treatments dramatically improve survival for women with HER2-positive breast cancer. Some women also get a second medication, pertuzumab, along with trastuzumab. It is also not clear whether the addition of pertuzumab to trastuzumab and chemotherapy impacts long-term survival rates or not [13].

This systematic review and meta-analysis aimed to assess whether adding pertuzumab to neoadjuvant chemotherapy and trastuzumab can improve the pathologic complete response (pCR) rates in early-stage HER2-positive breast cancer patients. Three double-arm clinical trials were identified that studied the effect of adding pertuzumab to trastuzumab to achieve a better pCR in early-stage HER2-positive breast cancer patients [14-16].

## Review

Methods

To ensure the quality of reporting, this study has been written in line with the Preferred Reporting Items for Systematic Reviews and Meta-Analyses (PRISMA) guidelines.

Inclusion and Exclusion Criteria

Only clinical trials were included in this study. Those studies were included that studied the pCR as their primary or secondary endpoint. pCR was defined as the proportion of patients without invasive cancer in the breast and axilla (ypT0/is and ypN0). Patients with HER2-positive breast cancer were included. The HER2-positive status was labeled as positive if the immunohistochemistry score was +3 or the fluorescence in situ hybridization (FISH) test score for HER2 was 2 or more than 2. Metastatic breast cancer patients were excluded from this study. Only those HER2-positive breast cancer patients were added who had operable, locally advanced, or inflammatory HER2-positive breast cancer. Patients in the neoadjuvant setting were only included. Adjuvant therapy outcomes were not included in this study. All cytotoxic chemotherapy regimens were considered eligible for this study, provided that the same drugs were given at the same dose in all study arms and that the study arms differed systematically only regarding pertuzumab administration.

If multiple publications of the same trial were retrieved or if there was a case mix between publications, only the most recent publication (and the most informative) was included.

Search Strategy

This study includes the articles retrieved from searching several databases. These databases were PubMed, Cochrane, and Embase, without year and language restrictions. Regarding the duration, the databases were searched for relevant articles published till May 2023.

The following search terms were used in PubMed: "breast neoplasms"[Mesh] AND ''chemotherapy''[Text Word] AND "trastuzumab"[Mesh] AND "receptor, ErbB-2"[Mesh]. We also searched Embase for relevant articles on our topic using keywords. Cochrane was also searched using the title, "Pathologic complete response observed in HER2 positive breast cancer patients."

Data Extraction

Two authors (FF and MA) were involved in data extraction from the three shortlisted clinical trials. Both authors extracted the data independently and agreed on all aspects of the final data that were retrieved in the Microsoft Excel file (Microsoft Corporation, Redmond, WA). The data from three studies were then extracted. Three studies were identified that studied the pCR to treatment by chemotherapy and trastuzumab vs. chemotherapy, trastuzumab, and pertuzumab. Data were extracted in the Microsoft Excel file based on pre-determined variables.

The data extracted from these three articles included the article title, DOI, authors, citation, publication year, number of participants, the median age of participants, clinical trials number, study design, the hormonal status of the tumor, staging of cancer, eligibility criteria, dosage detail of trastuzumab, dosage detail of pertuzumab, dosage of chemotherapy drug, the primary endpoint, a secondary endpoint, pCR, and conclusion of the study.

We also recorded, whenever possible, issues that reveal the quality of included studies: randomization model, allocation concealment, blindness, and withdrawal description.

Outcome Definition

The primary outcome of our study was the rate of pCR achieved. Two trials labeled pCR as the absence of residual invasive cancer in the breast and lymph nodes [15,16]. The third trial defined pCR as the absence of invasive neoplastic cells at the microscopic examination of the primary tumor at surgery [14]. Only one trial studied clinical response rate, time to clinical response, breast-conserving surgery rate, and safety as secondary endpoints [14]. The other two trails did not have any secondary endpoints [15,16]. Outcomes such as overall survival, event-free survival, and disease-free survival were not analyzed because these were not provided in any of the three trials and also because these outcomes were not a primary objective of this study.

Neoadjuvant Chemotherapy Regimens

Two trials included docetaxel (75 mg/m^2^), whereas the third one also included carboplatin. Neoadjuvant chemotherapy was given every three weeks along with targeted therapy till surgery.

Neoadjuvant Targeted Therapy Regimens

In all three double-arm trials included in this study, trastuzumab was given in both arms, but pertuzumab was administered in one arm in all three clinical trials. The primary purpose of adding trastuzumab + pertuzumab in one arm and only trastuzumab in the other arm was to observe the effect of adding pertuzumab in terms of achieving better pCR. The dose of trastuzumab was 8 mg/kg loading dose, followed by 6 mg/kg every three weeks. The dose of pertuzumab was 840 mg loading dose and 420 mg maintenance dose.

Statistical Methods

Data were analyzed using RevMan Web (Cochrane, London, UK). The odds ratio and 95% confidence interval were calculated for the outcome. The Mantel-Haenszel method and random effect model were used for analysis. Heterogeneity was assessed using I2, with I2 > 50% considered significant. The risk of bias in studies was evaluated using the Cochrane risk of bias tool for randomized controlled trials (ROB2).

Results

Selection of Studies

**Figure 1:**
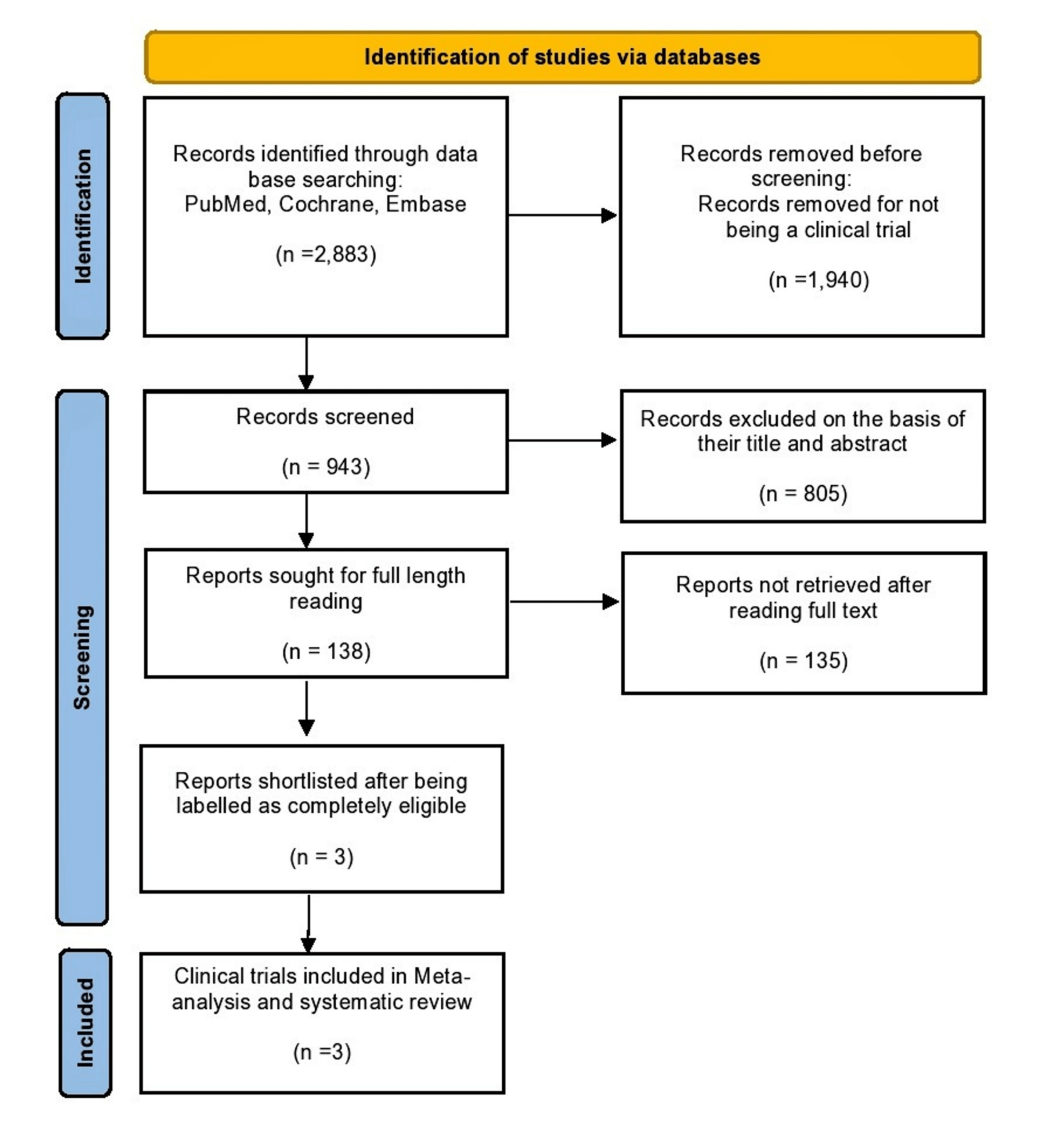
PRISMA flowchart of selection of studies PRISMA: Preferred Reporting Items for Systematic Reviews and Meta-Analyses.

2,328 studies showed up on PubMed, 165 studies showed up on Cochrane, and 390 studies showed up on Embase, making a total of 2,883 studies that were initially screened as shown in the PRISMA diagram in Figure 1. Out of these 2,883 studies, 1,940 studies were removed because they were not clinical trial studies. The remaining 943 studies were further screened, and 805 studies were excluded based on their title and abstract screening. The remaining 138 studies were sought for full-length read.

Of these 138 studies, only three were included in this study that strictly adhered to the inclusion criteria of our study. The rest of the studies were discarded after reading their full texts. A flowchart showing the study selection process is depicted in Figure 1.

Characteristics of Included Clinical Trials

Three clinical trials were included in this study. These three clinical trials were double-armed. One arm was based on targeted therapy with trastuzumab alone, another arm was based on targeted therapy with trastuzumab and pertuzumab. Pertuzumab was present in one arm while it was absent in the second arm, so the difference in pCR achieved after adding pertuzumab can be studied. The three double-arm trials included in this study compared the pCR in HER2-positive breast cancer patients after being treated with chemotherapy (C) + trastuzumab (T) + pertuzumab (P) vs. chemotherapy (C) + trastuzumab (T). In these studies, it was observed whether the addition of pertuzumab provided superior pCR compared to C + T alone. Characteristics of included clinical trials have been provided in Table 1.

**Table 1:** Characteristics of included clinical trials HER2: human epidermal growth factor receptor 2.

Study	Study design	Number of patients	Disease staging	Status of HER2	Neoadjuvant chemotherapy	Neoadjuvant trastuzumab and pertuzumab
Gianni et al. [14]	Multicenter, open-label, phase 2 study	107 in pertuzumab + trastuzumab + docetaxel and 107 in trastuzumab + docetaxel	Operable, locally advanced, or inflammatory	HER2 immunohistochemistry 3+ or 2+ and positive for fluorescence or chromogenic in situ hybridization	Docetaxel (75 mg/m²), escalating, if tolerated, to 100 mg/m² every 3 weeks	Trastuzumab (8 mg/kg loading dose, followed by 6 mg/kg every 3 weeks), pertuzumab (840 mg loading dose, followed by 420 mg every 3 weeks)
Shao et al. [15]	Multicenter, double-blind, placebo-controlled phase 3 trial	219 in trastuzumab + docetaxel + pertuzumab and 110 in trastuzumab + docetaxel	Early or locally advanced	HER2 immunohistochemistry 3+ or 2+ and positive for fluorescence or chromogenic in situ hybridization	Docetaxel (75 mg/m^2^) every 3 weeks	Trastuzumab (8 mg/kg loading dose and 6 mg/kg maintenance doses), pertuzumab (840 mg loading dose and 420 mg maintenance doses)
Beitsch et al. [16]	Multicenter, phase 2 study	119 in trastuzumab + docetaxel + carboplatin + pertuzumab and 178 in trastuzumab + docetaxel + carboplatin	T4 or inflammatory disease	HER2 immunohistochemistry 3+ or 2+ and positive for fluorescence or chromogenic in situ hybridization	Docetaxel (75 mg/m^2^), carboplatin every 3 weeks	Trastuzumab (8 mg/kg loading dose and 6 mg/kg maintenance doses), pertuzumab (840 mg loading dose and 420 mg maintenance doses)

Comparison of pCR Achieved in Experimental vs. Control Group

The summary statistics showed that the incidence of pCR was more in the experimental group as compared to the control group with an odds ratio of 2.10 (95% CI: 1.56-2.83) with I2 = 0%.

In three double-arm trials, there were 840 participants, 445 in the experimental group (C+T+P) and 395 in the control group (C+T). A total of 203 (45%) patients out of 445 in the experimental group achieved pCR, whereas 127 (32%) patients out of 395 in the control group achieved pCR.

In Gianni et al.'s (2012) study, 29% of the study population in the trastuzumab + docetaxel group achieved pCR. Whereas 45% of the study population in the trastuzumab + docetaxel + pertuzumab group achieved pCR [14].

In Shao et al.'s (2020) study, 21% of the study population in the trastuzumab + docetaxel group achieved pCR. On the other hand, 39% of the trastuzumab + docetaxel + pertuzumab group achieved pCR [15].

In Beitsch et al.'s (2017) study, 40% of the study population in the trastuzumab + docetaxel + carboplatin group achieved pCR. On the contrary, 57% of the study population in the trastuzumab + docetaxel + carboplatin + pertuzumab group achieved pCR [16].

**Figure 2:**
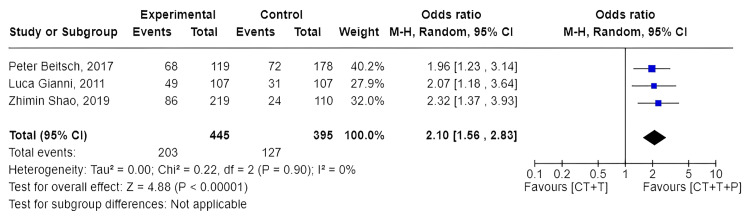
Forest plot comparing pathologic complete response of experimental and control groups Luca Gianni et al. (2012) [14], Zhimin Shao et al. (2020) [15], and Peter Beitsch et al. (2017) [16].

A forest plot depicting the pCR of these three double-arm trials is shown in Figure 2.

Summary of pCR

Table 2 provides the summary of pCR achieved in both arms of all three clinical trials. Endpoints and trial numbers of all three included clinical trials are also mentioned.

**Table 2:** Summary of pCR, endpoints, and trial numbers pCR: pathologic complete response.

Study	Definition of pCR	pCR, n (%)	Primary endpoint	Secondary endpoint	Trial number
Gianni et al. [14]	Absence of invasive neoplastic cells in the breast at the microscopic examination of the primary tumor at surgery	31/107 (29%) in trastuzumab + docetaxel and 49/107 (45%) in pertuzumab + trastuzumab + docetaxel	pCR	Clinical response rate, time to clinical response, breast-conserving surgery rate, and safety	NCT00545688
Shao et al. [15]	Absence of residual invasive cancer in the breast and lymph nodes	24/110 (21%) in trastuzumab + docetaxel and 86/219 (39%) in trastuzumab + docetaxel + pertuzumab	pCR	Not mentioned	NCT02586025
Beitsch et al. [16]	Absence of invasive carcinoma in both the breast and axilla at the microscopic examination of the resection specimen	72/178 (40%) in trastuzumab + docetaxel + carboplatin and 68/119 (57%) in trastuzumab + docetaxel + carboplatin + pertuzumab	pCR	Not mentioned	NCT01479101

Risk of Bias Graph

**Figure 3:**
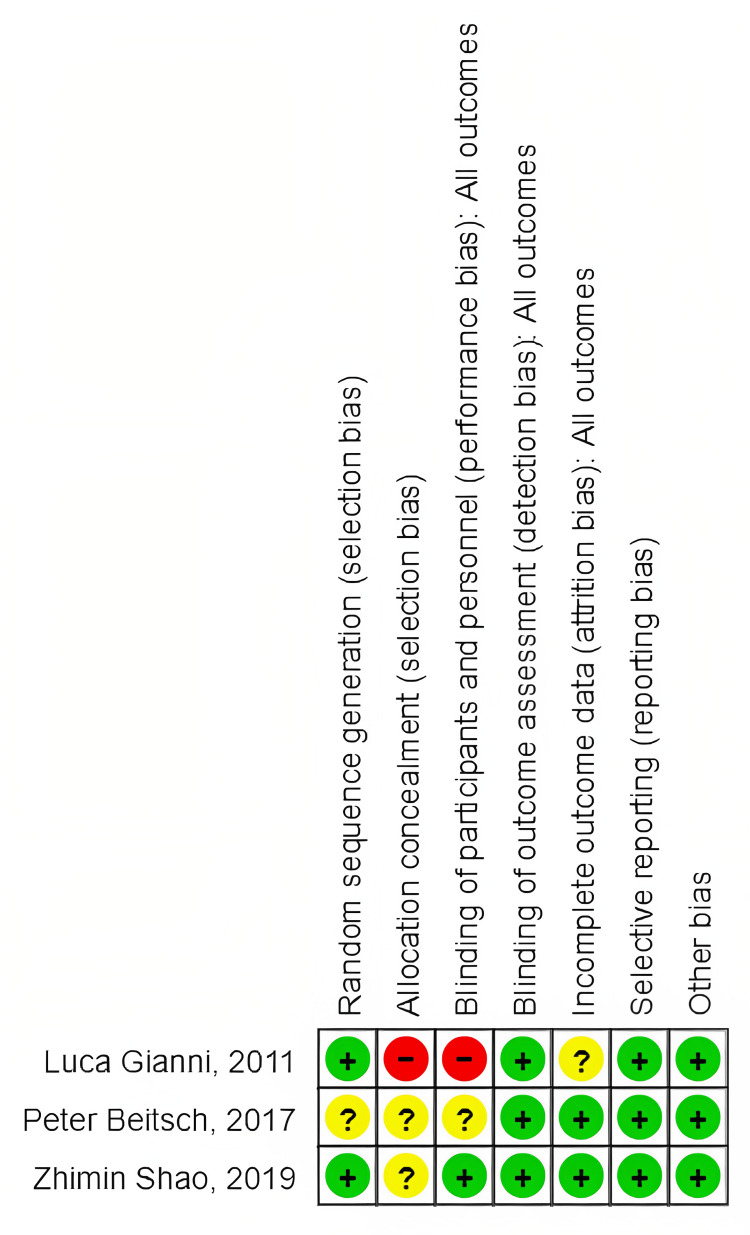
Risk of bias graph Luca Gianni et al. (2012) [14], Zhimin Shao et al. (2020) [15], and Peter Beitsch et al. (2017) [16].

The risk of bias graph is shown in Figure 3.

Risk of Bias Summary

**Figure 4:**
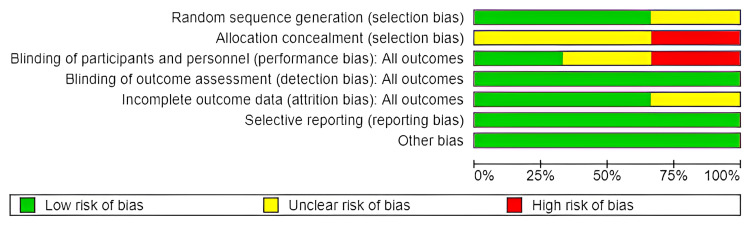
Risk of bias summary

The risk of bias summary is shown in Figure 4.

Discussion

This study shows that neoadjuvant treatment with chemotherapy, trastuzumab, and pertuzumab instead of chemotherapy and trastuzumab is of significant benefit in early-stage HER2-positive female breast cancer patients. All three clinical trials included and analyzed in this study suggest adding pertuzumab to the neoadjuvant treatment regimen. Individual results of all these three clinical trials also suggest having pertuzumab in the neoadjuvant settings [14-16]. A study conducted in the Netherlands on the data of 1124 patients showed that the addition of pertuzumab to chemotherapy and trastuzumab in the neoadjuvant settings resulted in achieving pCR in 65% of patients compared to pCR in only 41% who did not receive pertuzumab [17]. Another retrospective study conducted on 1528 female patients with locally advanced breast cancer showed that the pCR achieved in patients treated with chemotherapy, trastuzumab, and pertuzumab was more than the pCR in the group of patients who did not receive pertuzumab (66.4% vs. 56.8%, respectively; p < 0.001) [18].

Out of these three clinical trials included in this study, the highest pCR was achieved in Beitsch et al.'s (2017) trial. It was seen in the study population given a treatment regimen comprising trastuzumab + docetaxel + carboplatin + pertuzumab (TCHP) regimen. This might point out the usefulness of using this regimen in neoadjuvant settings of HER2-positive breast cancer patients. A retrospective study conducted in India showed that the TCHP regimen achieved pCR in 55.6% of the patients [19]. Another study in South Korea showed that patients given TCHP regimens in neoadjuvant settings achieved a pCR of 65% and a three-year event-free survival of 90% in real-world experience [20].

Adding pertuzumab to trastuzumab has also shown a relatively safe profile for breast cancer patients. A clinical trial incorporating trastuzumab and pertuzumab showed only modest side effects. This trial observed no death in patients given the neoadjuvant treatment, and grade 3 and 4 neutropenia and febrile neutropenia occurred in 12% and 8% of patients, respectively [21]. A retrospective study on pertuzumab, trastuzumab, and chemotherapy showed that this treatment regimen had a tolerable safety profile and that anemia was the most common adverse event (63.4%) observed in the study, and the most common grade 3-4 adverse event was nausea and vomiting (8.5%) [22]. A study on the safety and efficacy of pertuzumab in the treatment of HER2-positive breast cancer showed that although the most common side effect associated with the use of pertuzumab was diarrhea, most cases were not severe. The risk of pertuzumab-associated cardiac dysfunction was also low. In the same review, pertuzumab was not found to be cost-effective for patients [23].

A similar meta-analysis concluded that adding pertuzumab to trastuzumab resulted in a better overall survival rate than treatment with trastuzumab only. This meta-analysis observed the pCR rate as a secondary outcome. It concluded that the use of dual blockade (trastuzumab + pertuzumab) resulted in improved pCR rates compared to the use of mono blockade (trastuzumab only). This meta-analysis also concluded that the addition of pertuzumab to trastuzumab resulted in a significantly increased incidence of febrile neutropenia, diarrhea, and anemia [24]. Another meta-analysis involving trastuzumab, pertuzumab, and lapatinib showed that the rates of pCR rates for dual blockade (trastuzumab + pertuzumab) and mono blockade (trastuzumab only) were 51.60% and 38.26%, respectively. An improvement of 13.34% was seen in the pCR rate because of the addition of pertuzumab. The subgroup analysis of the pCR in the same meta-analysis showed that the regimen comprising trastuzumab + pertuzumab showed a more favorable pCR than the regimen comprising trastuzumab + lapatinib. The meta-analysis also depicted that adding pertuzumab to trastuzumab had no significant impact on cardiotoxicity [25].

An exploratory analysis of a few studies has shown that pertuzumab may be of no further benefit in small primary tumors or tumors that are node negative [23]. Thus, neoadjuvant treatment has to be individualized for each patient with HER2-positive breast cancer. The overall trend seen over the years still favors greatly the use of pertuzumab in addition to trastuzumab for the neoadjuvant treatment of early-stage breast cancer patients.

This study thus concludes that pertuzumab must be added to trastuzumab in the neoadjuvant settings in addition to the use of chemotherapy. Almost all previous clinical trials and retrospective studies suggest the use of pertuzumab in addition to the use of trastuzumab in neoadjuvant settings for HER2-positive breast cancer female patients to achieve a better pCR. Achieving pCR is associated with improved survival outcomes in the near and distant future for cancer patients. This fact has been supported by various retrospective studies mentioned in the discussion above and also by various meta-analyses conducted on the same topic as ours. Financial issues relating to the use of pertuzumab must also be addressed so that breast cancer patients can benefit from using pertuzumab without caring for the financial burden that might fall on them. The addition of pertuzumab to trastuzumab has not been shown to cause significant cardiotoxicity but some side effects may still be seen such as febrile neutropenia. These side effects must be considered while administration of pertuzumab and trastuzumab to these patients. Pertuzumab should be freely made available for patients with early-stage HER2 breast cancer to be used for breast cancer patients in neoadjuvant settings. The financial constraints must be addressed by the stakeholders so that eligible patients can receive pertuzumab without facing financial hurdles.

One of the limitations of this study is that only clinical trials were included in this study. No retrospective studies were included in the study. This is because we strictly wanted to see and analyze the results of clinical trials only. Another limitation is that survival outcomes were not studied in this study. Overall survival and progression-free survival were not studied and analyzed. One explanation for this limitation is that pCR is an indicator of survival outcomes, including overall and progression-free survival. Studying pCR only can predict the survival outcome in the distant future. Thus, this fact somewhat covers the deficiency caused by not studying the survival outcomes individually.

## Conclusions

This systematic review and meta-analysis concluded that pertuzumab should be added to trastuzumab as a targeted therapy for the neoadjuvant treatment of early-stage HER2-positive breast cancer patients. All individual clinical trials clearly show the benefit of achieving a higher pCR by adding pertuzumab in the neoadjuvant settings. Attaining higher pCR results in improved survival outcomes. Thus, dual-targeted blockade (trastuzumab + pertuzumab) should be considered the treatment of choice in neoadjuvant settings of early-stage HER2-positive breast cancer patients.
