# Correction: Pathologic Complete Response Achieved in Early-Stage HER2-Positive Breast Cancer After Neoadjuvant Therapy With Trastuzumab and Chemotherapy vs. Trastuzumab, Chemotherapy, and Pertuzumab: A Systematic Review and Meta-Analysis of Clinical Trials

**DOI:** 10.7759/cureus.c361

**Published:** 2025-10-15

**Authors:** Faizan Fazal, Muhammad Nauman Bashir, Maham Leeza Adil, Usama Tanveer, Mansoor Ahmed, Taha Zahid Chaudhry, Ali Ahmad Ijaz, Muhammad Haider

**Affiliations:** 1 Department of Medicine, Rawalpindi Medical University, Rawalpindi, PAK; 2 Department of Medicine, Holy Family Hospital, Rawalpindi, PAK; 3 Department of Surgery, Holy Family Hospital, Rawalpindi, PAK; 4 Department of Orthopedics, Holy Family Hospital, Rawalpindi, PAK

This article has been corrected to fix typos in the two paragraphs immediately preceding Figure 1 (PRISMA diagram). The original text included several typos resulting in data discrepancies between the text and PRISMA diagram. The PRISMA diagram displayed the correct data and the text has now been corrected to match.

